# Genome Sequences of *Lactobacillus* sp. Strains wkB8 and wkB10, Members of the Firm-5 Clade, from Honey Bee Guts

**DOI:** 10.1128/genomeA.01176-14

**Published:** 2014-11-13

**Authors:** Waldan K. Kwong, Amanda L. Mancenido, Nancy A. Moran

**Affiliations:** a Department of Ecology and Evolutionary Biology, Yale University, New Haven, Connecticut, USA; b Department of Integrative Biology, University of Texas, Austin, Texas, USA

## Abstract

We sequenced two strains from the *Lactobacillus* Firm-5 clade, a dominant group of symbionts in the guts of honey bees and other social bees. The genome of strain wkB8, comprising a 1.93-Mb chromosome and a 6.4-kb plasmid, was fully closed, while strain wkB10 was assembled into 32 contigs. These genomes will provide insights into how gut symbionts evolve and interact with their host species.

## GENOME ANNOUNCEMENT

Honey bees (*Apis* spp.) possess a highly specialized gut microbial community comprising about 8 bee-specific phylotypes (1). We recently sequenced the genomes of the major Gram-negative constituents, *Snodgrassella alvi* and *Gilliamella apicola* (2). Here, we present the genomes of two *Lactobacillus* strains of the Firm-5 clade, commonly identified as the most numerically abundant phylotype in honey bee guts (3, 4). This clade has also been reported in bumble bees (*Bombus* spp.) and stingless bees (*Meliponini*), which are close relatives of honey bees (5–7). Interestingly, host-associated *Lactobacillus* have not been found in other bee species, suggesting a uniquely coevolved symbiosis exists between Firm-5 and the social corbiculate bees (*Apis*, *Bombus*, and *Meliponini*) (8).

We isolated Firm-5 strains from the guts of the honey bee *Apis mellifera* as previously described (9), using Columbia agar with 5% sheep’s blood as a growth medium. DNA was extracted using phenol-chloroform and purified on DNeasy spin columns (Qiagen). Total genomic DNA was sequenced on the Illumina MiSeq platform from 2 × 250-bp paired-end libraries, returning 3,046,168 (strain wkB8) and 2,613,160 (strain wkB10) reads. Overlapping reads were combined using FLASH (10). All read types were then assembled with Velvet version 1.2.10 (11), producing a total of 18 and 32 contigs for wkB8 and wkB10, respectively. We successfully closed the wkB8 genome by *in silico* assembly inspection and combinatorial gap-spanning PCRs, although 45 ambiguous bases remain due to polymorphisms in the multicopy rRNA-encoding regions. Genomes were annotated with the RAST server (12).

The genome of wkB8 comprises a 1,926,135-bp chromosome and a 6,396-bp plasmid. It carries 1,772 predicted CDSs, 57 tRNAs, and 4 rRNA operons, and has a GC content of 36.7%. The wkB10 genome assembly was broken into 32 contigs with an *N*_50_ of 165,078, but appeared to represent a single chromosome. Examination of read coverage depth uncovered several contigs representing genomic regions likely present in multiple copies: contig018 (19 copies), contig019 (13 copies), and contig028 (5 copies). Thus, we estimate the wkB10 genome to be between 2.08 and 2.30 Mb in size, with 35.4% GC content. We detected 1,957 CDSs, 55 tRNAs, and at least 4 rRNA operons in wkB10. Both strains possess pathways to ferment various carbohydrates (e.g., fructose, lactose, mannose, *N*-acetylglucosamine, sorbose, sucrose, trehalose, xylulose) to lactic acid, and both encode a large number of predicted extracellular proteins that may allow adhesion to and degradation of environmental substrates such as chitin (13).

Strains wkB8 and wkB10 are 98.9% identical at their 16S rRNA locus but have only 86.0% average nucleotide identity across orthologous genomic regions. The bee-associated *Lactobacillus* Firm-5 cluster clearly comprises a diverse, deeply branching group of symbionts. Based on recent efforts to classify this group (14, 15), wkB8 is likely most related to *Lactobacillus helsingborgensis*, while wkB10 is part of *L. kullabergensis* or *L. kimbladii*; however, more genomes are required to unravel the murky relationships within the Firm-5 clade.

### Nucleotide sequence accession numbers.

The complete sequence of strain wkB8 has been deposited in GenBank under accession numbers CP009531 and CP009532, and the wkB10 whole-genome shotgun project has been deposited under accession number JRJB00000000.
